# Bioremediation of complex chlorinated-solvent impacted groundwater

**DOI:** 10.3389/fmicb.2026.1845514

**Published:** 2026-09-17

**Authors:** Evan Landers, Matthew Lee, Ranjith Rajendran, Emma Fay Harding, Katherine Picott, Mitra Parsa, Mike Manefield

**Affiliations:** 1 Department of Civil and Environmental Engineering, University of New South Wales, Sydney, NSW, Australia; 2 Chemical Engineering and Applied Chemistry, University of Toronto, Toronto, ON, Canada

**Keywords:** 1,2-dichloroethane, bioaugmentation, bioremediation, biostimulation, organohalide respiration, trichloromethane

## Abstract

Enhanced *in situ* bioremediation is a widely adopted and sustainable means of remediating chlorinated contaminant impacted groundwater utilizing organohalide respiring bacteria. These bacteria utilize chlorinated compounds as terminal electron acceptors to harvest energy, producing partially or fully dechlorinated end products. A barrier to successful remediation utilizing organohalide respiring bacteria is the impact of environmental factors which may be toxic or otherwise inhibitory towards these organisms or their respiration pathways, particularly in sites with complex contaminant profiles and heterogeneous geochemistry. In this study, groundwater from two regions of a heavily and mixed chlorinated solvent impacted site in Sydney, Australia with minimal historical evidence of natural attenuation was used to establish bench-scale microcosms for identification of factors that modulate successful bioremediation. The results of this study demonstrate that the success of enhanced bioremediation is minimally impacted by initial bacterial abundance with significant degradation of chlorinated hydrocarbons detected in microcosms with as low as 83 16S rRNA gene copies/mL following enhanced bioremediation, sulfate concentrations exceeding 71 mg/L may preferentially stimulate sulfate reducing bacteria, while chlorinated hydrocarbon concentration and composition remain the most inhibitory factors limiting bioremediation success.

## 1 Introduction

Bioremediation is an environmentally sustainable, socially acceptable and cost-effective strategy for mitigating pollution by harnessing the natural metabolic processes of microorganisms to degrade, transform, or sequester harmful contaminants. This approach is widely applied internationally as an alternative to conventional physicochemical remediation techniques, which often involve high energy demands, secondary pollution risks, and elevated costs (Mekonnen et al., 2024). Comparatively, bioremediation represents a more environmentally benign and economic means of achieving complete detoxification *in situ*, reducing the need for excavation or off-site treatment (Perez-Vazquez et al., 2024). Historically, bioremediation has been successfully implemented in the degradation of chlorinated aliphatic compounds in groundwater at high concentrations (Ellis et al., 2000; Hood et al., 2008). Advances in molecular microbiology, metagenomics, and biogeochemical modelling continue to improve the predictability and efficiency of bioremediation systems, reinforcing their potential as a core component in the sustainable management of organohalide contaminated environments (Yadav and Dharne, 2024). Among the various methods of bioremediation, natural attenuation—utilizing the remediation capacity of indigenous microorganisms without further intervention (Fan et al., 2022), biostimulation—the amendment of compounds that enhance the remediation capacity of indigenous microorganisms (Romantschuk et al., 2023), and bioaugmentation—the amendment of exogenous microorganisms to perform remediation activities (Xu et al., 2024a)—represent key approaches that differ in their mechanisms, implementation strategies, and suitability for various contamination scenarios.

Determination of which bioremediation method to choose necessitates characterization of the impacted site’s physiochemical properties and microbial communities. Sites lacking microorganisms that possess contaminant degradation pathways are unsuitable for natural attenuation (Davis, 2023). Likewise, indigenous microbial communities that possess pathways capable of remediation may be limited due to inhibition by co-contamination, nutrient availability, or competition from other microorganisms (Xu et al., 2023; Liang et al., 2021). All bioremediation methods require regular monitoring to assess changes in geochemistry, microbial population dynamics, and progress in contaminant remediation and management of these changes to ensure continued successful remediation and minimization of detrimental changes in the environment (Megharaj et al., 2011). While the fundamental parameters that drive the choice of bioremediation strategies are intuitive, the choice and viability of these strategies is complicated when applied to large dynamic heterogeneous systems, where multiple remediation approaches may be needed, or bioremediation is determined unviable.

In this study, the Botany Industrial Park (BIP) is used as model large (73 hectare) site showing heterogeneous groundwater physiochemistry and complex contaminant profiles. The BIP is situated in the southeastern suburbs of Sydney (New South Wales, Australia) and was a significant hub for chemical manufacturing from the 1940s to the 1990s. Due to poor historical waste disposal practices, the site has been heavily impacted by chlorinated solvents, with dissolved phase plumes extending several kilometers downgradient of the source zones threatening sensitive estuarian ecological systems and ultimately the waters of Botany Bay. The dominant class of contaminants at the BIP are comprised of chlorinated hydrocarbons (CHCs), including tetrachloroethene (PCE), trichloroethene (TCE), carbon tetrachloride (CT), trichloromethane (TCM), and 1,2-dichloroethane (1,2-DCA). The chemical properties of these compounds—high density relative to water and low solubility—make them persistent in the environment and prone to complex multiphase behavior in subsurface settings (Woinarski, 2022). The groundwater in the region is anoxic with low oxidation reduction potential (ORP) and is characterized by consistently low pH (<6) with pH decreasing at lower depths.

A variety of organohalide respiring bacteria (OHRB) have been discovered at this complex organochlorine impacted site. OHRB utilize chlorinated organic compounds as terminal electron acceptors for energy conservation. Two such OHRB species enriched from the site are *Desulfitobacterium* sp. AusDCA and *Dehalobacter* sp. UNSWDHB. *Desulfitobacterium* sp. AusDCA respires 1,2-DCA to ethene and is mildly acidotolerant with respiration detected at a pH as low as 5.0 (Low et al., 2019). *Dehalobacter* sp. UNSWDHB respires high (>100 mg/L) concentrations of TCM to DCM as well as respiring chlorinated ethanes including 1,1,1-TCA and 1,1,2-TCA (Wong et al., 2016). A third organism discovered from groundwater at the BIP, *Candidatus* Formimonas warabyi, while not an OHRB, ferments DCM to acetate (Holland et al., 2021). The presence of these organisms demonstrates that the microbial community at the site has been enriched for OHRB and driven to adapt to the presence of high concentrations of organochlorines. Additionally, these organisms are thought to be responsible for observed natural attenuation processes and can be used in bioaugmentation projects without incorporating exogenous organisms.

Despite the presence of OHRB, natural attenuation of chlorinated solvents remains minimal in some locations. This limited attenuation is likely driven by unfavorable physiochemical properties including low pH (often below pH 5) and elevated ORP which are broadly unfavorable to anaerobic metabolisms as well as contaminant levels/complexity in the aquifer that may inhibit the enzymatic activities of OHRB. Chlorinated solvent concentrations exceeding OHRB tolerance may be toxic to OHRB via inhibition of enzymatic activity or disruption of membrane integrity with concentrations of CT and TCM showing particular potency, inhibiting most OHRB activity at levels as low as 2 mg/L (Manefield et al., 2017). Loss of organohalide respiration may also occur indirectly with high solvent concentrations exerting toxic effects on microbes that provide OHRB with electron donors or essential vitamins (Wei et al., 2016). Moreover, the variety and heterogeneity of chlorinated compounds present in a site can influence organohalide respiration and microbial consortia structure, as different OHRB possess distinct reductive dehalogenases, which may be sensitive to organohalides other than their substrates (Richardson, 2016).

Understanding how environmental parameters such as pH and organochlorine diversity and load shape microbial communities is critical for the optimization of bioremediation strategies. Enhancing the activity and stability of OHRB through biostimulation or bioaugmentation requires a deeper understanding of microbial ecology and contaminant interactions (Major et al., 2002; Lu et al., 2021). In this study, two regions of the BIP with distinct physiochemical and contaminant profiles are investigated (Figure 1). The first region, termed the Nant region, is proximal and downgradient of the southern source zones and is heavily impacted by CT, its degradation product TCM; PCE, and its degradation product TCE as well as elevated levels of 1,2-DCA and VC. The Penrhyn region is downgradient of the Nant region and is impacted by contaminant mass from both the central and southern source zones with the predominant solvent being 1,2-DCA with elevated levels of TCM, PCE, TCE, and VC. Both regions have shown minimal evidence of natural attenuation since subsurface investigations were initiated in the 1990’s. However, between 2022 and 2023 dynamic changes in physiochemical and chlorinated solvent concentrations may indicate the regions are becoming more receptive to bioremediation strategies. This study aims to determine the impact physiochemical parameters and organochlorine contamination in the groundwater as of 2023 have on the microbial community at the BIP and how these parameters facilitate or inhibit bioremediation strategies that rely on organochlorine respiring bacteria.

**Figure 1:**
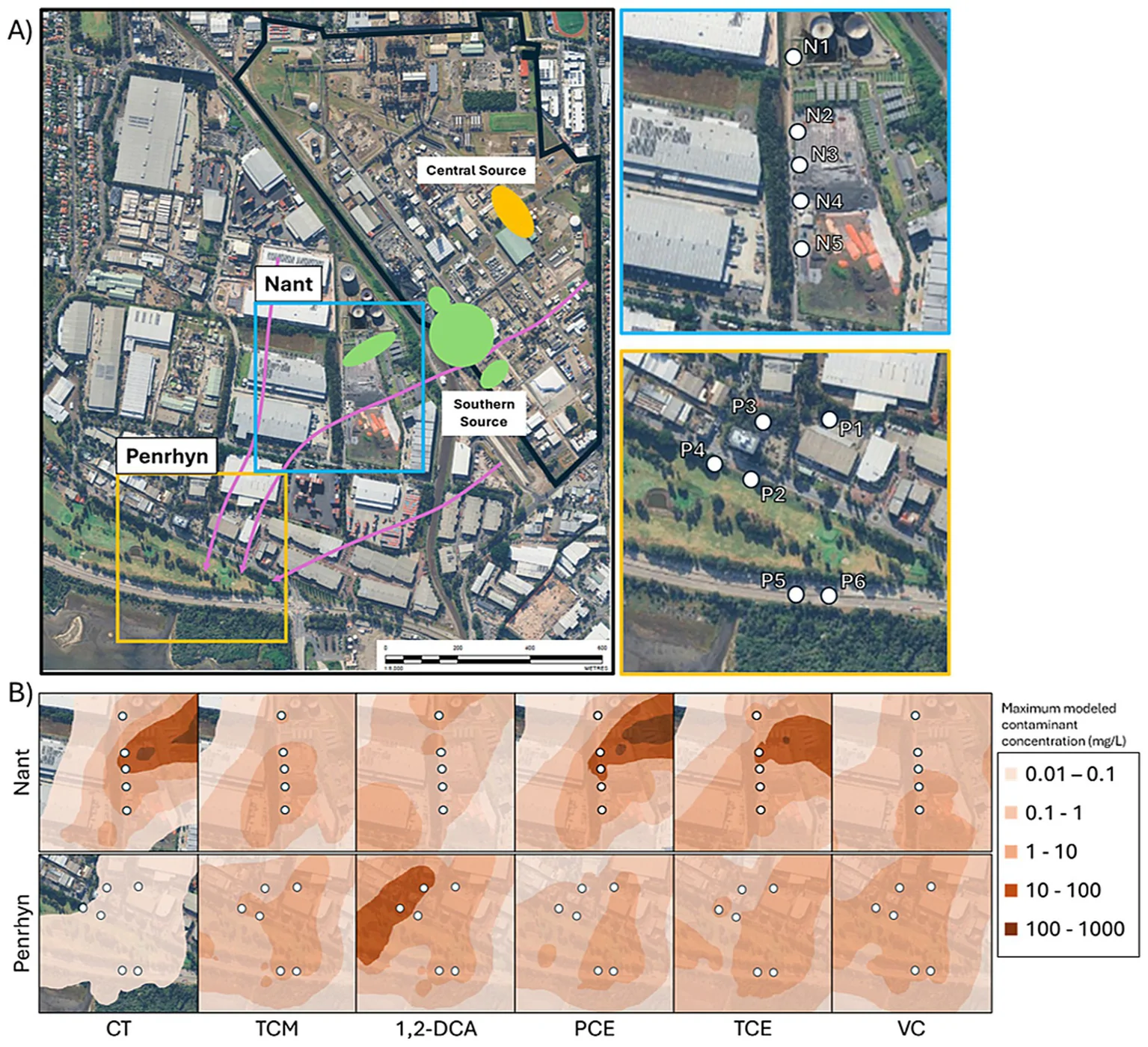
Map of impacted site and source locations of groundwater samples **(A)** Overview of Botany Industrial Park (black outline) and the Nant and Penrhyn regions. Orange and green regions indicate contaminant source zones; pink arrows indicate direction of groundwater flow. Well locations of groundwater used in this study are shown in magnified maps to the right. **(B)** Chlorinated hydrocarbon profiles of the groundwater in the Nant and Penrhyn regions; CT, carbon tetrachloride; TCM, trichloromethane; 1,2-DCA, 1,2-dichloroethane; PCE, perchloroethene; TCE, trichloroethene; VC, vinyl chloride. Map images generated using QGIS 4.1.

## 2 Materials and methods

### 2.1 Groundwater samples

Twenty-one groundwater samples from downgradient of the site were collected from groundwater monitoring wells in March 2023. Sampling locations were determined based on evidence of minimal natural attenuation. Six samples were from the Nant region, from five monitoring wells, N1 from a depth of 16 meters (N1-16), N2 from depths of 8 and 16 meters (N2-08 and N2-16), N3 from a depth of 14 meters (N3-14), N4 from a depth of 14 meters (N4 − 14), and N5 from a depth of 6 meters (N5-06). The remaining 16 groundwater samples were from six monitoring wells from the Penrhyn region: P1 at depths of 14, 18, and 20 meters (P1-14, P1 − 18, and P1-20), P2 from depths of 22 and 26 meters (P2-22 and P2-26), P3 from depths of 18, 22, and 26 meters (P3-18, P3-22, and P3-26), P4 from depths of 16 and 22 meters (P4 − 16 and P4-22), P5 from depths of 14 and 24 meters (P5 − 14 and P5-24), and P6 from depths of 8, 16, and 24 meters (P6-08, P6 − 16, and P6 − 24). Four liters of each sample were stored in 4 × 1 L glass bottles. All sample bottles had been purged with N_2_ prior to groundwater acquisition and were sealed with rubber septa (Schott) to maintain anaerobic conditions. Chemical profiles of samples were provided by remediation contractors.

### 2.2 DNA extraction of groundwater samples

Microbial biomass for initial community characterization was attained from 1 L groundwater by syringe filtration into 0.22 μm Sterivex filters (Millipore). Microbial biomass for community analysis of treated microcosms was collected from a single replicate (100 mL) of each treatment condition and likewise concentrated by syringe filtration into 0.22 μm Sterivex filters. The Sterivex filters were then amended with 1.5 mL of lysis buffer (50 mM Tris–HCl pH: 8.0, 40 mM Ethylenediaminetetraacetic acid (EDTA), and 0.75 M sucrose). Subsequently, 300 μL of freshly prepared lysozyme solution (125 mg/mL in molecular grade water) was added. The Sterivex filters were sealed, placed in 50 mL Falcon Tubes and incubated at 37 °C for 1 h. Following incubation, the samples were treated with 5 μL Proteinase K (20.0 mg/mL) and 100 μL 20% SDS. The tubes were rolled in an incubator at 120 RPM at 55 °C for 3 h to remove additional biomass on the filter and ensure complete lysis. The lysates were stored at −20 °C overnight.

Lysates were allowed to thaw on ice and 2 mL lysate was transferred to 15 mL Falcon tubes and mixed with an equal volumes of phenol:chloroform:isoamyl alcohol (25:24:1). The mixture was gently inverted 4–6 times and centrifuged at 2,500 rcf for 5 min. The aqueous phase was transferred to a new 15 mL Falcon tube. An equal volume of 7.5 M ammonium acetate (pH 8.0) was added to the aqueous phase, and the solution was mixed by inversion. Residual phenol was removed with the addition of 2 mL of chloroform, mixed by inversion (4–6 times), and centrifuged at 2,500 rcf for 5 min. The aqueous phase was aliquoted into three separate 1.5 mL Eppendorf tubes, each containing a final volume of 900 μL (total volume 2.7 mL). Ice-cold isopropanol (540 μL) was added to each tube (0.6 times aqueous volume) and mixed with vortexing. Samples were stored overnight at −20 °C to facilitate DNA precipitation.

Samples were centrifuged at maximum speed (15,000 rcf) for 20 min at 4 °C. The supernatant was removed by pipetting, and DNA pellets were washed with 300 μL of ice-cold 80% (v/v) ethanol. Samples were centrifuged again at 15,000 rcf for 10 min at 4 °C. The supernatant was removed by pipetting. DNA pellets were allowed to air-dry a biosafety cabinet for a minimum of 2 h. Dried DNA pellets were resuspended in DNase-Free water to a final volume of 40 μL. DNA quality and concentration was quantified using a Nanodrop™ One C spectrophotometer (ThermoFisher Scientific, Madison WI, USA).

### 2.3 Quantitative PCR (qPCR) of bacteria and metabolic genes

*Escherichia coli* DH5α stocks containing pCR 2.1 plasmids with insert sequences for the 16S rRNA gene from *Desulfitobacterium* for total bacterial 16S rRNA gene quantification (Munro et al., 2017), the *mcrA* gene for quantification of methanogenic archaea (Steinberg and Regan, 2008), and the *dsrA* gene for quantification of sulfate reducing bacteria (Kondo et al., 2004) (generously provided by Micronovo Technologies) were grown overnight in 5 mL Luria Bertani media (10 g tryptone, 5 g yeast extract, 10 g NaCl per liter). Plasmids were extracted from overnight cultures using the PureYield™ Plasmid Miniprep System, Promega following manufacturer’s instructions. Extracted plasmids were quantified by broad range Qubit 2.0 Fluorometer (Invitrogen). Copy numbers were determined using the formula:


$$\text{Copy Number}=\frac{[\text{Plasmid}\;\mathrm{DNA}]\mathrm{ng}*6.022*10^{23}}{(\text{Vector Length})*6.6*10^{11}\frac{\mathrm{ng}}{\mathrm{mol}}}$$

Where 6.022×10^23^ is Avogadro’s number, vector lengths correspond to plasmid size in bp, and 6.6×10^11^ ng/mol is the average mass of base paired nucleotides. Expected yields ranged from 10^10^–10^11^ copies/mL.

Quantified plasmid standards were serially diluted 1:10 in molecular grade water to generate qPCR standards ranging from 10^0^ to 10^7^ copy numbers per mL. DNA samples from groundwater samples were diluted 1:100 in molecular grade water. Samples and standards were run in 10 μL reaction mixtures containing 5.0 μL 2x SSoFastEvaGreen qPCR supermix (Bio-Rad), 0.1 μL each primer (Supplementary Table S1) (10 μM stock), 0.1 μL BSA (10 mg/mL), 2 μL DNA extract, and 2.7 μL molecular grade water. Reactions were run with an initial denaturation temperature of 95 °C for 2 min, followed by a denaturation step at 95 °C for 15 s, an annealing step at 51 °C for 30 s, and an elongation step at 72 °C for 30 s. Denaturation, annealing and elongation steps were repeated for a total of 40 cycles followed by a final elongation step at 72 °C for 2 min.

Samples were quantified in Bio-Rad CFX Maestro 1.0 using standard quantitation threshold values to generate a standard curve. Product accuracy was confirmed by examination of melting curve consistency across samples.

### 2.4 Community analysis of groundwater samples

Aliquots of 5–10 ng DNA extracts from groundwater samples were submitted to The Ramaciotti Centre for Genomics at the University of New South Wales (Sydney, NSW) for amplicon sequencing of the 16S rRNA gene V4 region using the Illumina MiSeq platform with v2 chemistry, generating paired-end reads of 2 × 250 bp. All reads used in this study can be found in the sequence read archive (SRA) under Bioproject PRJNA1510900. Raw FASTQ files were quality-checked and uploaded to the Galaxy platform (The GalaxyC, 2024), where they were organized into a collection for import into QIIME 2 v.2024.2 (Bolyen et al., 2019). Sequence data were imported using the QIIME tools import function, with the format set to “Casava 1.8 single-lane per sample.”

Denoising, chimera removal, and feature table construction were conducted using the DADA2 plugin (qiime dada2 denoise-paired). Reads were trimmed by 10 bases from the 5′ end of both forward and reverse reads (−-p-trim-left-f 10, −-p-trim-left-r 10) and truncated at 220 bp from the 3′ end of each read (−-p-trunc-len-f 220, −-p-trunc-len-r 220), retaining only high-quality sequence regions.

Representative sequences were aligned using MAFFT (QIIME alignment mafft), and the resulting alignment was masked to remove hypervariable and gapped regions using QIIME alignment mask. A phylogenetic tree was generated from the masked alignment with FastTree (QIIME phylogeny fasttree) and subsequently rooted using the midpoint-rooting method (QIIME phylogeny midpoint-root).

Taxonomic classification was performed using a Naive Bayes classifier trained on the SILVA 138.2 reference database using the QIIME feature-classifier fit-classifier-naive-bayes method. The classifier was then applied to the denoised representative sequences using QIIME feature-classifier classify-sklearn to assign taxonomy.

Alpha diversity rarefaction curves were generated with QIIME diversity alpha-rarefaction, using a maximum sampling depth of 7,000 sequences per sample to assess within-sample diversity saturation. Core diversity metrics were then computed with QIIME diversity core-metrics-phylogenetic, using a rarefaction depth of 3,000 sequences per sample. This analysis included both alpha diversity measures (e.g., Shannon diversity, observed features) and beta diversity metrics (e.g., weighted and unweighted UniFrac distances), leveraging the phylogenetic tree and taxonomy assignments.

### 2.5 Microcosm conditions

#### 2.5.1 Unamended

Unamended microcosms were established for all samples to determine the capacity of the groundwater to degrade CHCs via natural attenuation. For each groundwater sample, four replicates of 40 mL were transferred to 60 mL serum bottles that had been purged with N_2_ and sealed with Teflon coated rubber septa and aluminum crimp caps. A replicate of each groundwater sample was sterilized by autoclaving at 121 °C for 20 min to serve as an abiotic control. Unamended microcosms were monitored for 1,2-DCA and TCM degradation via GC-FID analysis.

#### 2.5.2 pH adjusted

Following the results of unamended microcosms, additional amendments were made to stimulate biological activity (summarized in Table 1). Three groundwater samples that seemed favorable to natural attenuation but were suspected to be inhibited by low pH alone were selected for pH adjustment. For pH adjusted microcosms, four replicates with 40 mL of groundwater samples P1-14, P2 − 22, and P5 − 24 were transferred anaerobically to sterile 60 mL serum bottles that had been purged with N_2_ and sealed with Teflon coated rubber septa and aluminum crimp caps. Groundwater samples had their pH assessed by Universal Test Paper Strips (Livingstone). The pH of each sample was adjusted to 7.0 with the addition of NaHCO_3_ (90 mg/mL). The headspace of each replicate was then pressurized with N_2_ gas to 0.5 bar. One replicate from each groundwater sample was autoclaved at 121 °C for 20 min to serve as an abiotic control. Microcosms that were pH adjusted only were monitored for 18 months prior to sacrifice for DNA extraction.

**Table 1:** Microcosm treatment index.

Condition	Code	Treatment
pH Adjusted	-pH	Adjusted pH to 7 with NaHCO_3_.
EVO Amended	-EVO	Adjusted pH to 7 with NaHCO_3_. Amended with EVO to final concentration of 0.001% v/v.
*Dehalobacter* Bioaugmented	-DHB	Adjusted pH to 7 with NaHCO_3_. Amended with EVO to final concentration of 0.001% v/v. Amended with 10 mL *Dehalobacter* sp. UNSWDHB enrichment culture.
*Desulfitobacterium* Bioaugmented	-DSB	Adjusted pH to 7 with NaHCO_3_. Amended with EVO to final concentration of 0.001% v/v. Amended with 10 mL *Desulfitobacterium* sp. AusDCA enrichment culture.

#### 2.5.3 pH adjusted and EVO amended

In replicates of four, 100 mL of all groundwater samples—N1-16, N2-8, N2-16, N3-14, N4-14, N5-6, P1-14, P1-18, P1-20, P2-22, P2-26, P3-18, P3-22, P3-26, P4-22, P5-16, P5-24, P6-08, P6 − 16, and P6-24 were transferred to sterile 120 mL serum bottles that had been purged with N_2_ and sealed with Teflon coated rubber septa and aluminum crimp caps. Samples had their pH adjusted to 7.0 with NaHCO_3_ as described above. Following pH adjustment, microcosms were amended with 1 mL of a 0.1% dilution of emulsified vegetable oil (EVO) made from canola oil with MicroEvo™ Self-Emulsifier (Tersus Environmental) to serve as a fermentable carbon and electron donor source (Harkness and Fisher, 2013). Microcosms were pressurized with N_2_ gas to 0.5 bar. One replicate from each groundwater sample was autoclaved at 121 °C for 20 min to serve as an abiotic control. Microcosms that were pH adjusted and amended with EVO were monitored for 15 months prior to sacrifice for DNA extraction.

#### 2.5.4 Bioaugmented

In replicates of four (N4-14, N1-16, P3-22, P4-22) or seven (P1-18, P3-26, and P6-08), 90 mL groundwater was transferred to sterile 120 mL serum bottle using 50 mL syringes (Becton Dickinson) to maintain anaerobic conditions. Each sample had its pH adjusted to a final pH of 7.0 with the addition of NaHCO_3_ as described above. Groundwater samples were then amended with either an enrichment culture of *Dehalobacter* sp. UNSWDHB (DHB) or an enrichment culture of *Desulfitobacterium* sp. AusDCA (DSB), to a final volume of 100 mL. One replicate from each groundwater sample was subsequently autoclaved at 121 °C for 20 min to serve as an abiotic control. Community composition of bioaugmentation enrichment cultures can be seen in Supplementary Figure S1—bioaugmentation culture community composition sequencing was performed in parallel with groundwater sample sequencing using bioaugmentation culture collected the same day as bioaugmentation microcosms were established. Microcosms that were bioaugmented were monitored for 10 months prior to sacrifice for DNA extraction.

### 2.6 Timepoint sampling and gas chromatography analysis

Microcosms were monitored by gas chromatography (GC) for ethene, VC, DCM, cDCE, TCM, 1,2-DCA, CT, TCE, PCE. Microcosms that were pH adjusted or pH adjusted with EVO were monitored every 3 months, while bioaugmented microcosms were monitored every month. Prior to sacrifice for DNA extraction, all samples were analyzed for a final time (month 18 for pH, 15 for EVO, and 10 for bioaugmented microcosms). Sampling was performed by transferring 1 mL of microcosm from each sample with a syringe to a 10 mL headspace vial (Agilent) and rapidly crimp sealing with a magnetic crimp cap. Injections were automated using an AOC-5000 Plus autoinjector (Shimadzu). Prior to injection, samples were heated to 80 °C for 3 min with shaking to equilibrate organohalide gas partitioning. Headspace gas was monitored on a Shimadzu GC-2010 Gas Chromatograph fitted with a flame ionization detector (FID) using a 30 m x 0.320 mm GS-Q column (Agilent Technologies). Gas chromatography was performed with an initial oven temperature of 150 °C, increasing at a rate of 30 °C per minute to a final temperature of 250 °C, with a hold time of 1.7 min. The injection was performed using a split inlet with a 10:1 ratio and a total carrier gas flow of 36 mL/min, with a split flow of 30 mL/min. The back detector was maintained at 250 °C with a hydrogen flow of 40 mL/min and an air flow of 450 mL/min. Samples were analyzed using GCsolution software (Shimadzu) using automated peak integration. Peak areas were converted to mg/L using standards generated from dilutions of pure stocks of organochlorines.

The three microcosm replicates were compared to the corresponding sterile control using one-sample t-tests at each time point. The sterile control value was treated as a fixed reference, with the test determining whether the mean of the three active replicates was distinguishable from this reference. The test accounted for variability between replicates via the standard deviation. Tests were conducted independently for each well and timepoint combination within each experimental condition. *p*-values were adjusted for multiple comparisons within each condition using Benjamini-Hochberg FDR correction (FDR; 5%). A result was considered significant when the active mean was below the sterile control and p_adj < 0.05. Microcosms that did not show significant differences between enhanced bioremediation conditions and abiotic controls were also analysed at an individual replicate level. Replicates reaching a 1,2-DCA and/or TCM concentration less than half of the abiotic control and remaining below that threshold for the duration of the monitoring period were considered to be showing ‘substantial’ chlorinated solvent degradation.

## 3 Results

### 3.1 Changes in groundwater characteristics are indicative of biological activity

The chemical profiles of 21 groundwater samples from the BIP; collected between 2/17–3/1/2023 were compared to the physiochemical and solvent profiles from corresponding groundwater sampling data taken a year prior (February 2022) to determine changes in physiochemical properties and how they may affect the microbial communities (summarized in Supplementary Table S2). Between 2022 and 2023, the average pH decreased from 5.47 to 5.04, with wells in the Penrhyn region showing a larger drop in pH (5.48 to 4.99) compared to those in the Nant region (5.44 to 5.15). Average ORP values were 73 mV lower across all samples in 2023 (−156 mV) than in 2022 (−83 mV), with a larger change in average ORP seen in Nant (−49 to −156 mV) as compared to Penrhyn (−95 to −155 mV). Sulfate concentrations decreased an average of 14.6 mg/L across all samples, with Nant showing a larger decrease in sulfate levels dropping from an average of 104 mg/L in 2022 to 64 mg/L in 2023; Penrhyn showed minor changes in sulfate concentrations dropping from an average of 48 mg/L to an average of 44 mg/L between 2022 and 2023. Individual samples showed larger shifts in sulfate concentrations with N2-8 and P2-22 decreasing by over 100 mg/L each, while P2-26 increased by 160 mg/L. Due to the ubiquity of sulfate reducing bacteria (SRB) under anoxic conditions, high sulfate concentrations may be indicative of limited biological activity under low redox conditions (Miao et al., 2012).

Chlorinated solvents across all wells consistently decreased between 2022 and 2023, apart from DCM and VC, which increased by an average of 0.054 and 9.21 mg/L, respectively. The average increase in DCM was driven almost entirely by P6-08, increasing from <0.005 to 2.22 mg/L (a higher concentration than all other wells combined). While an increase in VC was skewed by BP76-22 increasing by 186.6 mg/L, the average VC concentrations across all wells still increased slightly when P3-22 is excluded (1.59 mg/L in 2022 to 1.84 mg/L in 2023). Notably, both VC and DCM are products of organohalide respiration of cDCE and TCM, respectively; therefore, an increase in either of these may be indicative of active organohalide respiration. Between regions, Nant only showed an increase in VC, while Penrhyn showed an increase in PCE and cDCE in addition to DCM and VC. The increase in PCE is driven by wells P2-26 and P6-24, which increased from 0.196 to 4.76 mg/L and 3.83 to 8.87 mg/L, respectively. As PCE is not a product of organohalide respiration, these increases are likely driven by abiotic factors such as solvent desorption from DNAPL or plume migration—notably, concentrations of PCE in P2-22 drops by a similar level as it increases in P2-26, with P2-26 being 4 meters directly below P2-22 suggesting vertical migration.

Wells N2-08, N5-06 in the Nant region and P2-22, P3-22, and P5 − 14 in the Penrhyn region all showed decreases exceeding an order of magnitude in 5–7 solvents. These decreases along with decreases in sulfate concentration and ORP, are consistent with biotic activity and are more favorable to subsequent biological activity. However, these results do not eliminate the potential for abiotic effects driving these decreases such as migration events described above, solvent adsorption by substances in the aquifer matrix, or dilution by rainfall, with 2022 having experienced extensive rainfall (2,276 mm in the Port Botany region), with the months prior to extraction, January and February 2023, receiving 133.2 and 192.0 mm of rain, respectively, while historical averages for January and February are only 94.7 and 117.6 mm, respectively (bom.gov.au).

### 3.2 Differences in microbial profiles between sites is primarily driven by chlorinated solvent concentration and complexity

Bacterial abundance was quantified by qPCR of the 16S rRNA gene to determine the potential for microbial activity. Figure 2A shows the 16S rRNA gene copy numbers in the 21 groundwater samples. Average 16S rRNA gene copy number across all samples was 4.9×10^4^ copies/mL, while the Nant region averaged 3.5×10^2^ copies/mL and Penrhyn averaged 6.8×10^4^ copies/mL. Previous works had determined an average bacterial abundance across the BIP and Southlands as approximately 1×10^6^ copies/mL (Low et al., 2019; Munro et al., 2017), indicating the microbial community at these sites have been impacted, collectively having 20-fold less copies/mL, with the Penrhyn region showing 15-fold fewer copies/mL and the Nant region being more impacted with 2,854-fold fewer copies/mL.

**Figure 2:**
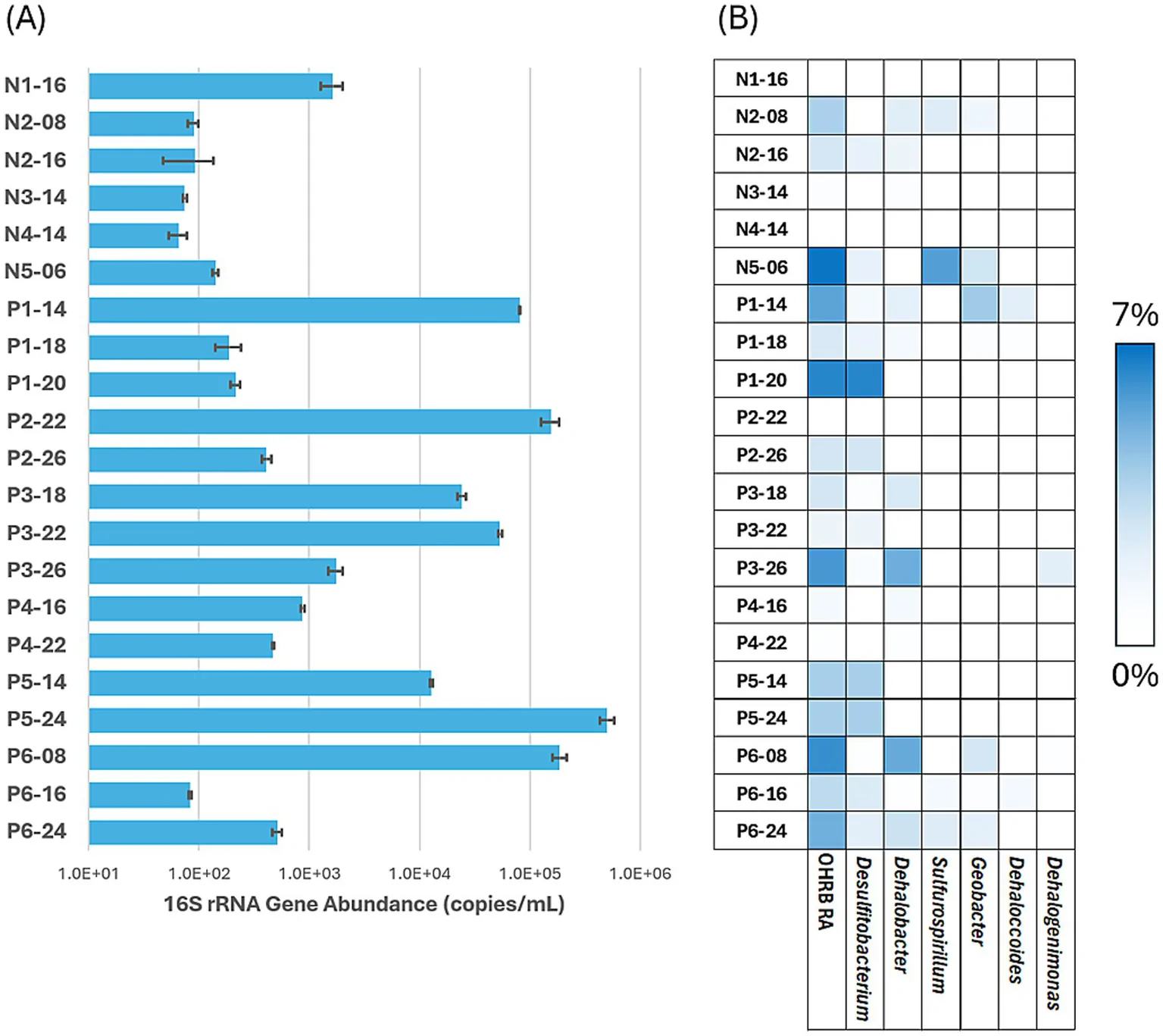
Microbial profiles of groundwater samples. **(A)** Total bacterial abundance in groundwater samples as determined by 16S rRNA gene qPCR, error bars represent standard deviation within technical replicates. **(B)** Heat map of relative abundance of total OHRB and by genera based on 16S rRNA gene sequencing.

As the abundance of bacteria was shown to be impacted at these sites, the relationship between 16S rRNA gene copy number and chlorinated solvent concentration was explored (Supplementary Figure S2). Broadly, concentrations of TCM, PCE, and TCE that exceeded 2 mg/L were associated with 16S rRNA gene concentrations of less than 10^3^ copies/mL; however, TCM concentrations also positively correlated with PCE and TCE concentrations (R^2^ = 0.79), thus the individual influence of these compounds on microbial abundance is difficult to distinguish from the influence of the others. Suppression of bacterial abundance correlated with higher concentrations of 1,2-DCA, with 16S rRNA gene copies below 10^3^/mL seen at 1,2-DCA concentrations of 30 mg/L. No significant correlation between total microbial abundance and cDCE was seen, suggesting concentrations exceeding those seen in these samples are required before inhibitory effects are seen. It is worth noting that the solvent concentrations at a specific location fluctuate, and the concentrations observed in this study offer a limited view of a dynamic system. Therefore, bacterial abundance may be influenced by historical solvent concentrations, secondary effects of solvents, or environmental factors unrelated to chlorinated solvent concentrations.

To determine the microbial capacity for anaerobic metabolisms with similar requirements as organohalide respiration in the groundwater samples, the functional genes involved in common anaerobic metabolisms—sulfate reduction (*dsrA,* the gene for dissimilatory sulfite reductase subunit A) from sulfate reducing bacteria (SRB) and methanogenesis (*mcrA*, the gene for methyl-coenzyme M reductase) from methanogenic Archaea—were quantified by qPCR (Supplementary Figure S3). The highest *dsrA* signals were identified in samples P3-22, and P5-24 at 378 and 508 copies/mL, respectively. The highest *mcrA* signals were observed in P3-18, P4-22, and P6-08 at 137, 141, and 572 copies/mL, respectively. All other groundwater samples showed low quantities (<100 copies/mL) of *dsrA* and *mcrA*.

The relative abundance of genera associated with characterized OHRB were examined to determine the potential of these samples to perform reductive dechlorination. Figure 2B shows the relative abundance of seven genera associated with organohalide respiration—*Desulfitobacterium, Dehalobacter, Sulfurospirillum, Geobacter, Dehalococcoides,* and *Dehalogenimonas* as determined by 16S rRNA community sequencing. Across all samples, OHRB represented 2.21% of the bacterial abundance on average, with the Nant region averaging 3.26% and the Penrhyn region averaging 1.8%, with N5-6 from the Nant region showing the highest relative abundance at 6.06%. Across all samples, *Desulfitobacterium* and *Dehalobacter* genera were the most abundant at 0.81 and 0.66% total relative abundance. Samples P3-22 and P3-26 lacked any representative OHRB genera despite P3-26 having the highest total CHC load.

### 3.3 Viable enhanced bioremediation strategies are predicted to be site-specific

Initial work examined the biological natural attenuation capacity of the groundwater samples via monitoring transformation of chlorinated solvents in unamended microcosms relative to sterile controls. Only a single groundwater sample—P1 − 14—from the Penrhyn region showed bioactivity (Supplementary Figure S4). Degradation of 1,2-DCA was seen as early as 30 days after microcosm establishment, with complete degradation of 1,2-DCA seen after 60 days. After 8 months of monitoring, no other unamended microcosm showed evidence of degradation of organochlorines present, consistent with long-term (decades) persistence of the contaminants at the site.

As most samples were shown incapable of natural attenuation, bioactivity markers were used to classify groundwater samples in terms of their likelihood to respond to enhanced bioremediation (biostimulation or bioaugmentation). Table 2 summarizes these results separating ‘enhancing’ traits—traits that are indicative of bioactivity or supportive of biological activity—and ‘inhibitory’ traits—traits that are indicative of reduced bioactivity or would prevent biological activity. Traits categorized as enhancing include evidence of dechlorination between 2022–2023, bacterial abundance, presence of organohalide respiring genera, solvent concentrations below inhibitory levels, and pH above 5.0. Inhibitory traits include low bacterial abundance (<10^3^ cells/mL), chlorinated solvent concentrations above inhibitory levels, sulfate above 80 mg/L, carbon disulfide concentrations exceeding 1 mg/L, elevated sulfate concentrations, and pH below 5.0. The number of inhibitory traits was subtracted from the number of enhancing traits, with high bacterial abundance, high relative abundance of OHRB, and low bacterial numbers being more heavily weighted, being direct measures of bioactivity. Scores greater than 3 were considered bioactive (white), scores between 0–3 were considered potentially bioactive (light gray), and scores less than 0 were considered solvent impacted (dark gray). Bioactive samples are expected to respond favorably to biostimulation, potentially bioactive samples were those where the outcome of either biostimulation or bioaugmentation for remediation was possible, but more uncertain, and solvent impacted samples were not expected to respond to either bioremediation strategies without a reduction in current solvent concentrations. In the Nant region, two samples were categorized as bioactive, one was considered potentially bioactive, while the remaining three were all categorized as solvent impacted. The Penrhyn region had eight samples categorized as bioactive, two as potentially bioactive, and five as solvent impacted. Based on this predictive model, groundwater samples were biostimulated and/or bioaugmented to determine if groundwater conditions were amenable to bioremediation using these techniques. All microcosms had their pH adjusted to ~7.0 to facilitate OHRB activity (Microcosms ending in “-pH” were only stimulated by pH adjustment). Following pH adjustment, biostimulation microcosms were amended with EVO to provide fermentable substrates for fermentative bacteria to produce electron donors (e.g., hydrogen) to be utilized by OHRB for organohalide respiration (Microcosms ending in -EVO). Bioaugmented microcosms were likewise pH adjusted and amended with EVO, as well as amended with 10 mL of either a culture of *Dehalobacter* sp. UNSWDHB (DHB) or *Desulfitobacterium* sp. AusDCA (DSB). Microcosms were monitored for degradation of 1,2-DCA and TCM.

**Table 2:** Summary of bioactivity in groundwater samples.

Expected effect	Environmental parameter	N1-16	N2-08	N2-16	N3-14	N4-14	N5-06	P1-14	P1-18	P1 − 20	P2 − 22	P2 − 26	P3-18	P3 − 22	P3 − 26	P4-16	P4 − 22	P5-14	P5 − 24	P6-08	P6-16	P6 − 24
Enhancing	Field CF reduction (DCM > 0.1 mg/L)			+1					+1			+1				+1	+1		+1	+1	+1	+1
	Field PCE reduction (PCE + TCE)/(DCE + VC) < 1		+1				+1	+1			+1		+1	+1	+1	+1	+1	+1		+1	+1	
	Bacterial Abundance (16S) > 10^3^	+1						+1			+1		+1	+1	+1			+1	+1	+1		
	Bacterial Abundance (16S) > 10^4^							+1			+1		+1	+1				+1	+1	+1		
	Bacterial Abundance (16S) > 10^5^										+2								+2	+2		
	OHRB Relative Abundance > 1%	+1	+1		+1	+1	+1			+1	+1	+1	+1			+1	+1	+1	+1		+1	+1
	OHRB Relative Abundance > 2%		+1			+1	+1			+1	+1	+1	+1						+1		+1	+1
	OHRB Relative Abundance > 3%		+2			+2	+2			+2		+2									+2	
	OHRB Absolute Abundance > 10^2^							+1					+1	+1				+1	+1	+1		
	OHRB Absolute Abundance > 10^3^							+1											+1	+1		
	Solvents < 25 mg/L		+1				+1	+1			+1		+1	+1		+1	+1	+1		+1	+1	
	pH > 5		+1				+1	+1	+1		+1					+1		+1		+1	+1	
Inhibitory	Solvents > 25 mg/L	−1		-1	-1	-1			-1	-1		-1			-1				-1			-1
	Solvents > 50 mg/L			−2					-2	-2		-2			-2							-2
	CF > 2 mg/L			−1	−1	−1			−1	−1		−1										−1
	1,2-DCA > 50 mg/L								−1	−1		−1			−1							−1
	Chlorinated ethenes > 20 mg/L			−1	−1	−1									−1							−1
	Sulfate > 80 mg/L			−1	−1	−1				−1		−1										−1
	CS_2_ > 1 mg/L			−1	−1	−1																−1
	pH < 5	−1		−1	−1	−1				−1		−1	−1	−1	−1		−1		−1			−1
	Bacterial Abundance (16S) < 10^3^		−2	−2	−2	−2	−2		−2	−2		−2				−2	−2				−2	−2
	Balance	0	5	−9	−7	−4	5	7	−5	−5	9	−4	6	4	−4	3	1	7	7	10	6	−8

Enhancing conditions are likely to support bioremediation and were scored as +1 or +2. Inhibitory conditions are likely to inhibit bioremediation and were scored −1 or −2. Scores for each sample were totaled, scores less than −1 were considered unlikely to respond to bioremediation (dark gray), scores greater than 3 were considered likely to respond to bioremediation (white) and scores between −1 and 3 were considered potentially responsive to bioremediation (light gray).

The results of these incubations are summarized in Table 3 (solvent concentration data for all conditions can be found in Supplementary Tables S3–S5, and Supplementary Figures S5–S8) While the predicted bioactivity of 14 of the microcosms unambiguously corresponded to the actual bioactivity seen, there were also many notable exceptions between predicted and experimental results. Of the 32 cultivation conditions, 5 showed significantly greater degradation of 1,2-DCA compared to abiotic controls (*p* < 0.05) in all replicates, and 6 showed significantly greater (p < 0.05) degradation of TCM than abiotic controls in all replicates. Replicates were also identified which showed substantial degradation (concentrations less than half of the starting 1,2-DCA and/or TCM concentrations) were also seen, even when other replicates in that condition showed no significant changes compared to abiotic controls. Ten microcosms had replicates displaying these substantial degradation profiles—six degrading 1,2-DCA, two degrading TCM, and two degrading both 1,2-DCA and TCM. Six groundwater samples that were predicted to respond favorably to enhanced bioremediation showed degradation of at least one of the chlorinated solvents in one or more treatment conditions; however, four groundwater samples predicted to respond favorably to enhanced bioremediation (N5-06, P3-22, P3-26, and P5 − 14) showed no evidence of solvent degradation in any replicate. Of the four groundwater samples predicted to possibly respond to bioremediation, only P6 − 16 showed bioactivity when amended with EVO in a limited number of replicates (replicates 1 and 2) after 9 months of incubation. Intriguingly, of the samples predicted to not respond to bioremediation, two microcosms from these samples showed degradation of at least one organochlorine in one or more replicate Microcosms from P1 − 18 showed significant degradation of 1,2-DCA after 1 month when amended with *Desulfitobacterium,* significant TCM degradation after 1 month when amended with *Dehalobacter* sp. UNSWDHB, and substantial degradation of 1,2-DCA or TCM in some replicates of EVO amended microcosms after nine months. Likewise, groundwater from P3-26 showed significant degradation of TCM when amended with *Dehalobacter* sp. UNSWDHB after 1 month.

**Table 3:** Results of enhanced bioremediation.

	Degradation Detected	
Microcosm	1,2-DCA	TCM
N1-16-EVO	–	–
N2-08-EVO	R1 (12); R3 (12)	All (6)
N2-16-EVO	–	–
N3-14-EVO	–	–
N4-14-EVO	–	–
N5-06-EVO	–	–
P1-14-pH	All (3)	R1 (12)
P1-14-EVO	All (3)	R2 (9); R3 (6)
P1-18-EVO	R2 (9)	R3 (9)
P1-18-DHB	–	All (1)
P1-18-DSB	All (1)	–
P1-20-EVO	–	–
P2-22-pH	R1 (12)	R1 (15)
P2-22-EVO	All	–
P2-26-EVO	–	–
P3-18-EVO	R3 (9)	–
P3-22-EVO	–	–
P3-26-EVO	–	–
P3-22-DSB	–	–
P3-26-DHB	–	All (1)
P3-26-DSB	–	–
P4-16-EVO	–	–
P4-22-EVO	–	–
P4-22-DSB	–	–
P5-14-EVO	–	–
P5-24-pH	–	–
P5-24-EVO	R2 (3)	All (15)
P6-08-EVO	R1 (6)	All (15)
P6-08-DHB	R3 (2)	–
P6-08-DSB	All (1)	All (2)
P6-16-EVO	R1 (9); R3 (9)	–
P6-24-EVO	–	–

Samples indicated well location, depth and treatment condition: pH, pH adjusted; EVO, pH adjusted + EVO; DSB/DHB, pH adjusted, EVO amended, and augmented with Desulfitobacterium, or Dehalobacter, respectively. Color of sample indicates previously established prediction of remediation: White, likely receptive to bioremediation; Light Gray, potentially responsive to bioremediation; Dark Gray, Unlikely to respond to bioremediation. ‘Degradation detected’ columns indicate if degradation of either 1,2-DCA and/or TCM was seen in that condition. ‘All’ indicates significant (*p* < 0.05) degradation was detected in all replicates relative to abiotic control–green for 1,2-DCA, blue for TCM; R1, R2, and R3 indicate a decrease in CHC concentration to less than half of the abiotic control was seen in replicate 1, 2, and/or 3, respectively, but not in all replicates, orange for 1,2-DCA, yellow for TCM. Numbers in parentheses indicate the earliest time point of the incubation that differences were detected.

### 3.4 Heavy and mixed chlorinated hydrocarbon contamination limits bioremediation capacity

One of the major factors considered in determining the expected bioremediation capacity of the groundwater samples was the high CHC load in the samples. Additionally, particularly in the Nant region, the composition of the total CHC was complex, containing a mixture of chlorinated ethenes, ethanes, and methanes. Initial estimates showed that concentrations of 1,2-DCA exceeding 30 mg/L were inhibitory towards biological activity, while concentrations of TCM as low as 2 mg/L inhibited biological activity. To determine how accurate these initial estimates were, the CHC concentrations and compositions of samples that responded favorably to bioremediation compared to those that failed to demonstrate bioremediation were examined (Figure 3).

**Figure 3:**
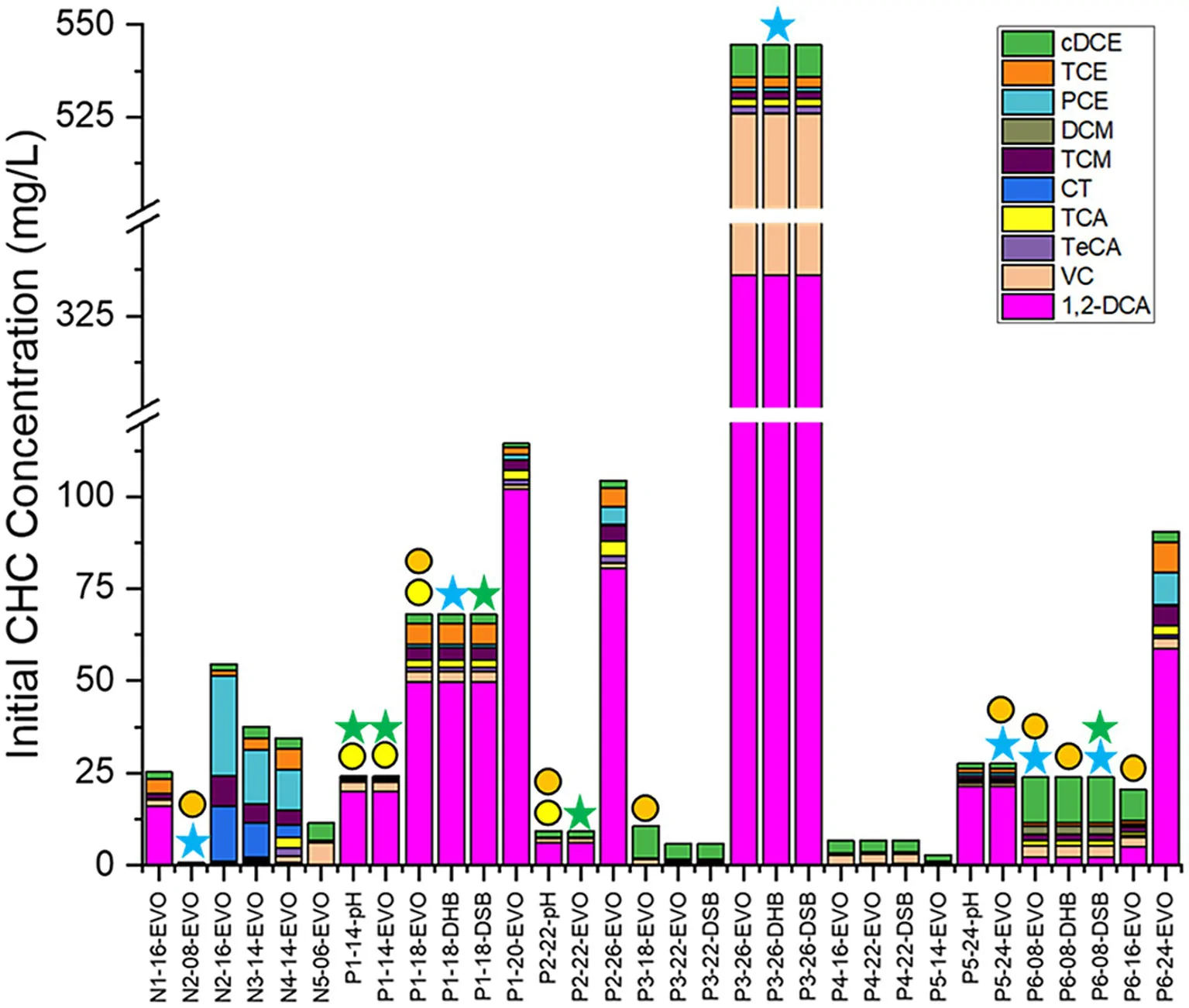
Initial chlorinated hydrocarbon impact on biostimulated or bioaugmented microcosms. Concentrations of chlorinated hydrocarbons present in initial groundwater samples; cDCE, 1,2-*cis*-dichloroethene; TCE, trichloroethene; PCE, perchloroethene; DCM, dichloromethane; CT, carbon tetrachloride; TCA, trichloroethanes; TeCA, tetrachloroethane; VC, vinyl chloride; 1,2-DCA, 1,2-dichloroethane. Shapes above bars indicate the outcome of bioremediation cultures: Star, remediation successful in all replicates (green, 1,2-DCA, blue, TCM); Circle, bioremediation successful in one or two replicates (orange, 1,2-DCA, yellow, TCM).

Broadly, lower CHC concentrations corresponded to increased activity. The average CHC concentration of a microcosms displaying degradation of chlorinated solvents was 63.1 mg/L (28.7 mg/L without P3-24-DHB), while microcosms that failed to show any activity had an average CHC concentration of 95.6 mg/L. Notably, microcosms from P1 − 18 showed degradation of 1,2-DCA and/or TCM, despite groundwater concentrations of 1,2-DCA exceeding 30 mg/L, and TCM concentrations exceeding 2 mg/L; however, based on measurements of the groundwater over the course of these experiments, these values were both less than their respective initial values (1,2-DCA in P1 − 18 ranged from 25–30 mg/L, while TCM ranged from 1–2.2 mg/L).

Intriguingly, microcosoms from P1 − 18 groundwater showed degradation of both 1,2-DCA (P1 − 18-EVO replicate 2 and P1-18-DSB) and TCM (P1 − 18-EVO replicate 3 and P1-18-DHB) but in no individual microcosm showed degradation of both solvents. Groundwater from P3-26 had the highest total CHC concentrations exceeding 500 mg/L total; however, the *Dehalobacter* sp. UNSWDHB augmented microcosm was able to degrade all TCM (0.5 mg/L) after only one month. Of the microcosms from the Nant region (N1-16-EVO, N2-08-EVO, N2-16-EVO, N3-14-EVO, and N5-06), only N2-08-EVO—the least solvent-impacted sample amongst all samples—showed activity, degrading 1,2-DCA in two replicates and TCM in all replicates. All other microcosms from the Nant region were likely inhibited by the complexity of the CHC composition, particularly high concentrations of chlorinated ethenes and TCM paired with low starting OHRB abundances.

### 3.5 Enhanced bioremediation can function in low cell-density regions

Prediction of bioremediation capacity for these samples was, in part, driven by the abundance of bacteria present in the samples. Generally, bacterial abundance is a good approximation of how favorable a site is to bacterial activities, as environments that cannot support bacteria naturally have low bacterial abundance. To determine how well bacterial abundance acts as a predictor of bioremediation capacity, initial 16S rRNA gene copy numbers were compared to bioremediation outcomes of this study. When comparing the starting bacterial abundance to how successful a site was for remediation (Figure 4A), it can be seen bacterial abundance is a relatively poor predictor of bioremediation capacity. Conditions with significant chlorinated solvent degradation or replicates with substantial successful remediation had an average starting bacterial community of 1.04×10^5^ cells/mL, while microcosms that failed to demonstrate any level of chlorinated solvent degradation had initial bacterial abundances averaging 3.73×10^4^, more than a third of the successful average. Furthermore, biostimulation was seen in microcosms with 16S rRNA gene copy numbers as low as double digits in N2-08-EVO and P6 − 16-EVO at 89 and 83 copies/mL, respectively. The microcosms from P5-24, which had the highest initial bacterial abundance at 5.06×10^5^ copies/mL, failed to show bioremediation activity when only pH adjusted, while EVO amendment required 15 months for significant TCM degradation and only showed substantial 1,2-DCA degradation in a single replicate. Collectively, these results demonstrate that bacterial abundance alone is not a strong indicator of how successful a biostimulation or bioaugmentation approach would be at a given site.

**Figure 4:**
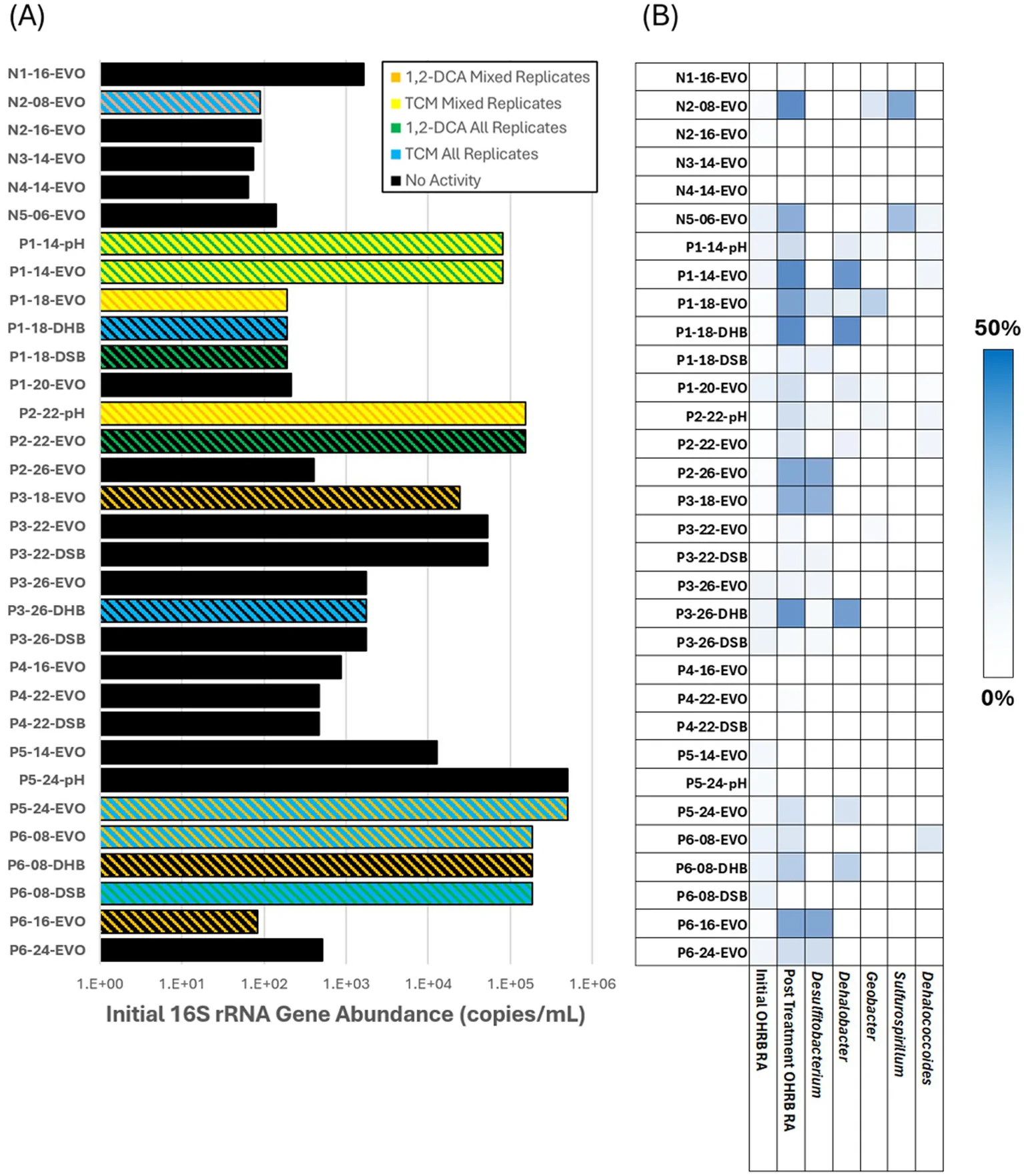
Bacterial profiles of bioremediation microcosms. **(A)** Starting bacterial abundance as determined by 16S rRNA quantification. Bar colors correspond to remediation outcomes for 1,2-DCA and TCM degradation in either all, some, or no replicates; error bars represent standard deviation between technical replicates. **(B)** Heatmap of relative abundances of OHRB initially detected in groundwater samples, OHRB in samples following enhanced bioremediation, and OHRB genera in samples following enhanced bioremediation. Only OHRB genera at >2% RA are shown.

To determine how bioremediation strategies affected the OHRB populations, the relative abundances of known OHRB genera were calculated based on their abundances in community analyses. Of the known OHRB genera, only five were detected at relative abundances exceeding 2%: *Desulfitobacterium, Dehalobacter, Geobacter, Sulfurospirillum,* and *Dehalococcoides.* Other genera capable of organohalide respiration of CHC found at the BIP—*Dehalogenimonas, Desulfomonile,* and *Dechloromonas*—were also detected, although their relative abundances only account for 0.02–0.26% of their communities. Notably, organisms belonging to the *Desulfovibrio* genus were also abundant in many of these microcosms; however, as these organisms are only known to be facultative OHRB, and, to date, are not known to respire either 1,2-DCA nor TCM, it is most likely these organisms are acting as SRB in these microcosms and are therefore not included as OHRB.

Microcosms from less organochlorine impacted groundwater became enriched for OHRB following biostimulation or bioaugmentation (Figure 4B). The average OHRB abundance increased from an average of 2.66% in initial groundwater samples to 16.22% in biostimulated or bioaugmented microcosms, with three microcosms (N2-08-EVO, P1-14-EVO, and P1-18-DHB) being nearly 50% of the total bacterial community. Only eight microcosms showed a decrease in OHRB relative abundance (N2-16-EVO, N3-14-EVO, P3-26-EVO, P3-26-DSB, P4-16-EVO, P5-14-EVO, P5-24-pH, and P6-08-DSB), with seven of those also showing a lack of bioremediation. Intriguingly, of the eight that showed a decrease in OHRB relative abundance, two of them were bioaugmentation microcosms that had been amended with *Desulfitobacterium*, and—in the case of P6-08-DSB—the microcosm had shown activity and a loss of both 1,2-DCA and TCM.

This trend of low relative abundance of *Desulfitobacterium* is seen throughout the bioaugmentation microcosms. Of the microcosms amended with *Desulfitobacterium* sp. AusDCA, the highest relative abundance seen is only 7.0% in P1-18-DSB, while two microcosms lacked any detectable *Desulfitobacterium* (P4-22-DSB and P6-08-DSB). In contrast, four of the EVO amended microcosms (P2-26-EVO, P3-16-EVO, P6-16-EVO, and P6-24-EVO) all showed relative abundances of *Desulfitobacterium* exceeding 10%, with all but P6-24-EVO exceeding 30% relative abundance; however, only two of the high abundance *Desulfitobacterium* microcosms actually resulted in bioremediation, both of which were only seen in a subset of the experimental replicates. These changes in relative abundance may not necessarily indicate a loss of *Desulfitobacterium* sp. AusDCA, as potential increases in the absolute abundances of all microbes in these cultures may mask a healthy, but not dominant, *Desulfitobacterium* sp. AusDCA population. Based on sequencing, the majority of *Desulfitobacterium* seen was the same species as AusDCA, with *Desulfitobacterium* sp. CR1 being present at high abundance in P1-18-EVO (6.9%—more than twice the abundance of *Desulfitobacterium* sp. AusDCA in the sample), and at low abundances in P2-22-EVO (0.2%) and P5-24-EVO (0.6%). *Desulfitobacterium* sp. PR was also detected at low abundances in P3-18-EVO (0.1%) and P3-26-DHB. Collectively, this suggests biostimulation and bioaugmentation of these groundwaters predominantly results in the enrichment of *Desulfitobacterium* sp. AusDCA.

Microcosms bioaugmented with *Dehalobacter* sp. UNSWDHB in contrast all had high (>30%) relative abundances of *Dehalobacter* following treatment. Two *Dehalobacter* species were detected by sequencing; *Dehalobacter* sp. UNSWDHB, and an uncultured *Dehalobacter* species that is presumably native to the BIP groundwater. Outside of bioaugmentation microcosms (including N1 − 16-DSB and N4-14-DSB) *Dehalobacter* sp. UNSWDHB was only detected at low abundance (0.4%) in P1-20-EVO. The uncultured *Dehalobacter* species was responsible for the presence of *Dehalobacter* in all microcosms that were not amended with *Dehalobacter* sp. UNSWDHB and was also present at elevated relative abundances concurrently with *Dehalobacter* sp. UNSWDHB in P3-26-DHB (11.3%) and P6-08-DHB (4.6%).

*Geobacter* and *Sulfurospirillum* were each represented by a single species. Two *Dehalococcoides* lineages were present, neither of which could be resolved to a species level.

### 3.6 Sulfate reducing bacteria are co-enriched through remediation

All microcosms that had initial sulfate concentrations exceeding 71 mg/L resulted in no detectable CHC degradation. The presence of sulfate is used to predict the bioactivity of an environment. Elevated levels of sulfate are associated with reduced bioactivity, as the presence of SRB should decrease sulfate levels by reducing sulfate to less oxidized forms, with SRB being key organism in microbial networks (Wasmund, 2023). This criterion was part of the groundwater assessment. To determine if initial sulfate concentrations were a reliable predictor of bioremediation capacity, the initial sulfate concentrations of the groundwater were compared to their capacity to degrade 1,2-DCA and TCM (Figure 5).

**Figure 5:**
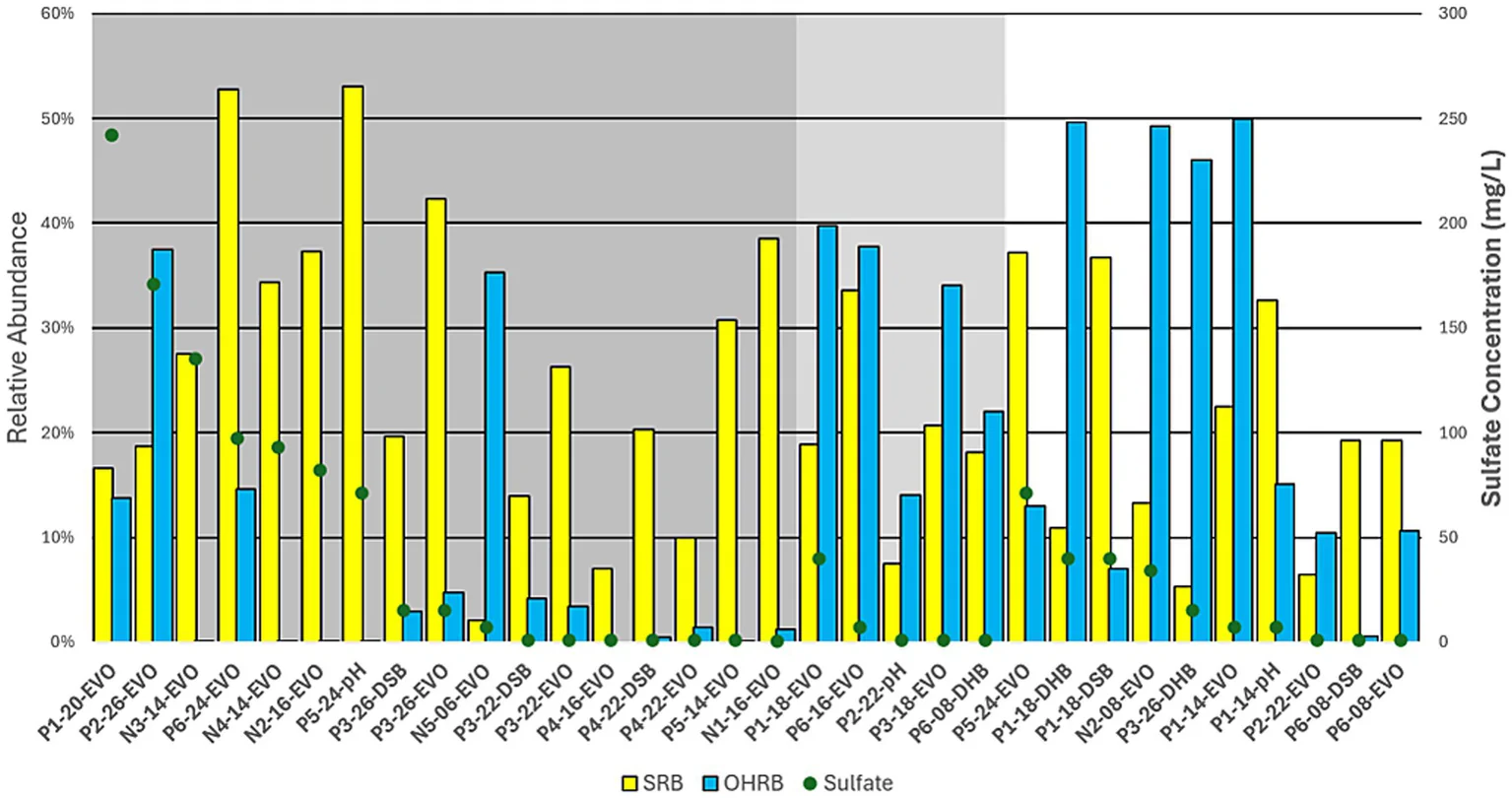
Relative OHRB and SRB abundances after bioremediation cultivation. Yellow bars represent the relative abundance of SRB, and blue bars represent the relative abundance of OHRB after remediation experiments. Green dots indicate the initial sulfate concentration in each groundwater sample (mg/L). Background color corresponds to outcome of bioremediation culture: Dark gray, No bioremediation; Light gray, Remediation of 1,2-DCA and TCM was limited to some replicates but not all; White, Remediation of 1,2-DCA and/or TCM was seen in all replicates for at least one solvent.

The highest initial sulfate concentration of the microcosms that showed significant CHC degradation was 71 mg/L from microcosm P5-24-EVO; the average sulfate concentration of microcosms showing any level of successful 1,2-DCA or TCM degradation was 17.8 mg/L. Microcosms that failed to demonstrate biodegradation of 1,2-DCA or TCM had an average sulfate concentration of 55.0 mg/L. Biostimulation of these samples also resulted in the enrichment of SRB. Members of *Desulfurella*, *Thermodesmonas*, *Desulfosporosinus, Desulfatirhabdium,* and *Caldisericum* were all enriched to greater than 2% relative abundance in at least one microcosm. The average SRB abundance in microcosms that did not demonstrate degradation of chlorinated solvents (27.3%) was higher than in those that were able to degrade 1,2-DCA and/or TCM (19.8%). Collectively, these results indicate that sulfate alone is not a strong indicator of a site’s bioremediation capacity; however, sites with sulfate concentrations exceeding ~80 mg/L are likely unfavorable to bioremediation or may in a greater lag time before degradation of CHCs is seen. These results also indicate that biostimulation and bioaugmentation of sites may lead to preferential enrichment of SRB over OHRB—whether subsequent bioaugmentation treatments would overcome this limitation requires further study.

## 4 Discussion

### 4.1 Gaps in chemical profiles and microbial communities should be addressed for wholistic monitoring practices

As the activity of OHRB is influenced by the physiochemical and microbial populations of their environment, it is worth considering factors not accounted for in this study as potential mediators of reductive dechlorination. Chemically, many factors were not directly measured which may have had an impact on OHRB in the microcosms. Metabolic byproducts of other members of microbial communities in the microcosms (even microbial populations which may have been transient) could have impacted the OHRB populations. Hydrogen sulfide has been shown to inhibit OHRB at levels exceeding 0.1–1 mM (Mao et al., 2017). Based on the prevalence of sulfate and SRB in the groundwater used in this study, it is possible hydrogen sulfide accumulated because of SRB activity—a potential alternative to direct competition with SRB. If hydrogen sulfide is acting to inhibit OHRB, this may be negligible in actual groundwater where more diffusion is possible; additionally, ferrous iron has been demonstrated to mitigate this effect by scavenging hydrogen sulfide to form ferrous sulfide (Lu et al., 2026). The ferrous iron concentrations in the groundwater and/or aquifer matrix may therefore self-modulate the impact of hydrogen sulfide *in situ*. Similarly, nitrous oxide has been shown to be a potent inhibitor of OHRB activity (Yin et al., 2019). It is more difficult to estimate the levels of nitrous oxide in the groundwater as direct measurements have not been made to our knowledge, with most measurements of the groundwater’s nitrogen concentrations focusing on nitrates and nitrites. Historical usage of fertilizers may have led to an increase in nitrous oxide following ammonium oxidation, while the presence of nitrates in the groundwater may lead to their reduction resulting in nitrous oxide.

Biologically, two major groups that may play an important role in *in situ* bioremediation work were not accounted for in these studies: the bacteriophage community and aquifer-matrix associated organisms. Bacteriophages are prevalent throughout the biosphere and play important roles in regulating bacterial populations via infection and subsequent cell lysis. Bacteriophages also contribute to biodiversity through transfection providing mechanisms for the movement of genetic material and subsequent attributes between bacteria (Hernández and Vives, 2020; Muniesa et al., 2013). Identification of bacteriophages that target organisms that compete with OHRB may lead to their subsequent use in bioremediation. Alternatively, identifying regions enriched with indigenous OHRB-infecting bacteriophages may shape which taxa of OHRB are appropriate at a given site. Future studies that explore bacteriophage populations and their influence on the BIP microbiota would be informative in understanding additional biotic factors that can impact bioremediation success. Organisms associated with the aquifer matrix can impact bioremediation work and should be considered. Groundwater sampling from monitoring wells is limited to organisms that are free-living or transiently mobile, therefore any populations that adhere to solid surfaces either directly or as part of biofilm communities, will not be detected through groundwater sampling methods. As it is hypothesized that the majority of environmental bacteria exist in biofilms, it is probable there are substantial biofilm-associated communities in the subsurface which are omitted from this study due to the source material used (Flemming and Wuertz, 2019). Previous work examining aerobic bioremediation of 1,4-dioxane and TCE saw differences in functional gene abundances when comparing microbial communities present in groundwater samples to those found in Bio-Traps®, with a gene for phenol hydroxylase detected at several orders of magnitude greater abundance in Bio-Traps®, while a ring hydroxylating toluene monooxygenase gene was orders of magnitude higher in groundwater samples (Chiang et al., 2012). Additionally, many organisms benefit from syntrophic interactions to enhance metabolic processes that may be inhibited or limited outside of biofilm communities (Brileya et al., 2014; Pillot et al., 2020). It is worth noting that attempts to extract DNA from excavated aquifer matrix did not result in quantifiable DNA (data not shown). Thus, it may be that the BIP aquifer matrix—or at least the regions heavily impacted by chlorinated solvents—lack significant biofilm communities. Although it is also possible the extraction process itself (sonic drilling), may have impacted these biological communities by acting similarly to bead beating—a common experimental method for cell lysis.

### 4.2 Predictive measures of biological remediation capacity

Previous predictive models for determining the bioremediation capacity of contaminated sites predominantly focused on sites’ capacities for bioremediation utilizing *Dehalococcoides* species for dechlorination of chlorinated ethenes (Lee et al., 2016; Di Curzio et al., 2024); however, despite the known capacity of *Desulfitobacterium* and *Dehalobacter* to reduce organochlorines, less work has focused on the environmental conditions that facilitate their capacity for dechlorination (Maphosa et al., 2012). Several lines of evidence indicate that biological dechlorination is occurring at the BIP. The observed decreases in ORP and sulfate concentrations between 2022 and 2023 are consistent with conditions favorable to reductive dechlorination (Smits et al., 2004; Hermon et al., 2019). The reduction in heavily chlorinated solvents in many wells, along with corresponding increases in their respiration products like VC and DCM, supports a biotic transformation pathway. However, the accumulation of VC in P3-22 and the increase in DCM in P6-08 are consistent with incomplete reductive dechlorination. A limited number of OHRB are known to respire DCE and VC to ethene, and even those that can frequently show accumulation of VC (Maymó-Gatell et al., 2001; DiStefano et al., 1991). Likewise, anaerobic DCM degradation is predominantly fermentative, with no organism, to our knowledge, capable of DCM respiration (He et al., 2003; Holland et al., 2021).

The presence of SRB and methanogenic Archaea, along with their functional genes (*dsrA*, *mcrA*), indicates that anaerobic bioactivity is present, with SRB, OHRB, and methanogens often requiring similar environmental parameters. However, the interaction between SRB, methanogens, and OHRB is complex. There is evidence that SRB and methanogens can outcompete OHRB for electron donors, especially under substrate-limited conditions, which may potentially inhibit dechlorination activity (Mao et al., 2017; Nuzzo et al., 2017). These competitive interactions may explain some of the variability observed in dechlorination efficacy across wells. However, there is also recent evidence of beneficial relationships between OHRB and SRB, particularly in the presence of heavy metalloids where they have been shown to help facilitate dechlorination of TCE by *Dehalococcoides* species (Li and Zhao, 2025). How the specific species of SRB, methanogens, and OHRB under the conditions of each well at the BIP interact remains to be elucidated.

Detection of OHRB in this study was performed by community analysis by 16S rRNA sequencing and identification of genera known to perform organohalide respiration. This approach does rely on the assumption that the members of the detected genera are capable of organohalide respiration. While members of some genera like *Dehalococcoides* are obligate OHRB (Yang et al., 2020), other genera such as *Desulfitobacterium* have members that lack the genetic capacity to respire CHC (Kruse et al., 2017). As an alternative, detection of *rdhA* genes—the genes that encode the reductive dehalogenase proteins directly involved in reductive dehalogenation of CHCs—would more directly show the genetic capacity of a community to respire chlorinated solvents. This method is complicated by the tendency of OHRB to possess multiple *rdhA* genes, many of which are not known to be expressed (Hug et al., 2013). Additionally, under certain environmental conditions, facultative OHRB may be enriched without contributing to functional reductive dechlorination—an inherent complication in both this study as well as one assessing *rdhA* gene abundances. While the predictive measures used in this study were comparatively simple and focused on a small number of biologically relevant parameters, more advanced methods such as machine learning may be instrumental in determining site remediation viability. Machine learning models have already been generated for a handful of bioremediation strategies including for BTEX and antibiotic degradation as well as heavy metal sequestration (Zhang et al., 2023; Sun et al., 2022; Kamal et al., 2024). Due to the large historical dataset that has been generated as part of the groundwater monitoring program at the BIP, there is potential for the generation of a machine learning model based on the site. Incorporation of successful and unsuccessful remediation results as developed in this study could then be incorporated into that model to determine which parameters underpin the success of a given remediation strategy. Provided a viable model is generated this way, ongoing monitoring of the site could be used to target regions that are prime candidates for bioremediation at a given time point, with subsequent successes or failures providing feedback to inform and refine the model.

### 4.3 High chlorinated solvent concentrations limit biological remediation capacity

The most prominent limiting factor seen in this study was the inhibition of microbial degradation of CHCs in locations with high CHC load. This is consistent with previous works exploring the effects of high concentrations of halogenated compounds as driving factors in decreased microbial abundance and diversity (Herrero et al., 2022). Additionally, the effect of mixed organochlorines was shown to result in less successful remediation than in samples with a lower number of different organochlorines. This is consistent with previous works in which microbial populations exposed to mixed contaminants were shaped in distinct ways when exposed to complex contaminant profiles compared to individual contaminants (Tang et al., 2022). There are several ways to address the limitations on bioremediation success due to organochlorine load and complexity: abiotic treatments to decrease CHC loads, targeting lower CHC density regions, and/or approaching the contaminants from a hierarchy.

Abiotic removal of CHCs is ongoing at the BIP, as the groundwater treatment plant continues to remove CHC from the groundwater. Additional abiotic options that are feasible include the use of chemically induced dechlorination. Abiotic reductive dechlorination can be performed with compounds such as zero-valent iron (ZVI), which has been demonstrated to reduce chlorinated ethenes, potentially all the way to ethene (Taghavy et al., 2010). Radical-induced oxidation using compounds such as Fenton’s reagent (H_2_O_2_ + Fe^2+^) or persulfate (S_2_O_8_^2−^) can oxidize CHC, leading to their mineralization (Seol and Javandel, 2008; Xu et al., 2024b). High density CHCs can also be reduced by sorption onto introduced compounds such as activated carbon or biochars, thereby lowering the dissolved phase concentration of CHCs. From the perspective of long-term bioremediation, ZVI represents the most attractive of these options as the other two options have the potential to be detrimental to bioremediation. Introduction of radicals into systems can be lethal to microbes, particularly in anaerobic environments where organisms are more likely, on average, to be sensitive to chronic oxidative stress (Lu and Imlay, 2021). However, ablation of native microbial populations by oxidative treatments could potentially be leveraged for subsequent bioaugmentation. In this scenario, recolonization of the groundwater by amended bioaugmentation cultures would proceed with reduced competition from native microbes potentially enhancing the success rate of bioaugmentation. This approach would necessitate that the groundwater and aquifer matrix is not rendered toxic or otherwise uninhabitable following chemical oxidation. Sequestration of CHCs by sorption has shown mixed results, it has been shown to reduced bioavailability of the CHC to the microbial system with evidence of a decrease in the Chloroflexi community seen in some studies (Ali et al., 2019), while others have reported increased organochlorine degradation following the introduction of biochar, albeit under aerobic conditions (Gregory et al., 2015).

The second approach, targeting areas with lower concentrations of CHC, effectively ‘eating the plume from the outside’ would target regions amenable to bioremediation (such as those which resulted in successful outcomes in this study) first. Ideally, the organisms would degrade low density regions and migrate toward higher density regions gradually as local CHC concentrations decreased. This approach does necessitate that remediating organisms are capable of migration toward the contaminant, either actively or passively. Previous studies examining the motile bacterium *Pseudomonas putida* G7’s degradation of polycyclic aromatic hydrocarbons, showed motility increased the capacity of *P. putida* G7 to access sorbed hydrocarbons for degradation (Rolando et al., 2020). Members of *Desulfitobacterium* and *Dehalobacter* have been characterized as being motile via flagellation, although chemotaxis studies would be required to determine what stimuli their motility apparatuses respond to specifically (Wong et al., 2016; Gerritse et al., 1996; Villemur et al., 2006; Tang et al., 2016).

The third option is to address the contaminants from a hierarchy of bioremedial inhibition. It has been shown that the presence of one (or more) organochlorine species can have inhibitory effects on the degradation of other organochlorine species (Futagami et al., 2013). In this study it was seen that TCM and PCE were both more inhibitory than 1,2-DCA on general microbial activity as well as bioremediation. Therefore, biological degradation of 1,2-DCA may be dependent on the prior degradation of TCM and/or PCE to concentrations 1,2-DCA degrading bacteria can tolerate. Additional insights into the complex relationships between contaminants and OHRB will be required to fully formulate a strategy to remove contaminants sequentially. Although some of the relations seen in this work already demonstrate potential paths forward. As seen in P3-26-DHB, *Dehalobacter* sp. UNSWDHB can degrade TCM even at high concentrations of 1,2-DCA and PCE. This demonstrates TCM can be removed even in high concentrations of mixed CHC, subsequent treatment of TCM-removed P3-26 groundwater may show it to be amenable to PCE degradation, which then in turn may result in groundwater amenable to 1,2-DCA degradation. It is worth noting this activity was on TCM at a concentration of approximately 0.5 mg/L—so whether this activity would be present with higher concentrations of TCM in similarly complex and regions with high concentrations of CHC bears further examination. Notably, *Dehalobacter* sp. UNSWDHB has been demonstrated to respire, and is regularly cultivated on, TCM at concentrations exceeding 100 mg/L (Wong et al., 2016). As TCM is typically inhibitory on OHRB respiration at concentrations of 2 mg/L or less, *Dehalobacter* sp. UNSWDHB is uniquely suited to handling high TCM concentrations and, as demonstrated in this study, can dechlorinate TCM in the presence of 1,2-DCA concentrations exceeding 100 mg/L. Alternatively, it may be feasible that sites with complex CHC mixtures may be receptive to simultaneous bioaugmentation with multiple OHRB capable of respiring the CHC present (e.g., amending a region with 1,2-DCA and TCM with both *Desulfitobacterium* sp. AusDCA and *Dehalobacter* sp. UNSWDHB). In this scenario, any OHRB which are not inhibited by the compounds present may respire their respective CHC to the point that the activity of other OHRB is no longer inhibited, leading to the degradation of other CHCs. This does necessitate that the CHCs are only inhibitory (i.e., bacteriostatic) and not lethal for the organisms introduced. Ultimately, based on the complexity and scale of the BIP groundwater contamination, an integrated approach of abiotic and biotic remediation approaches will likely be required.

Beyond decreasing chlorinated solvent concentrations, it is also worth considering the conditions bioaugmentation is implemented. *In situ* bioaugmentation using OHRB necessitates an environment that is amenable to organohalide respiration. The amendment of EVO in this study was largely to stimulate fermentative bacteria to generate H_2_ and other potential electron donors for organohalide respiration. The application of EVO at low (unadjusted) pH was not addressed in this study but may be a viable biostimulation approach. Provided acidophilic and/or acidotolerant OHRB are present, the amendment of an electron donor may be sufficient to stimulate organochloride respiration independent of pH adjustment. Furthermore, adjustment of pH, amendment of EVO, and addition of bioaugmentation cultures was performed simultaneously in this study; however, there is evidence that sequential treatment can enhance bioremediation. Wu et al. (2023) demonstrated the pretreatment of samples from a chloroethene contaminated site with a culture of fermentative bacteria and methanogenic archaea 4 days prior to amendment with a *Dehalococcoides* culture for PCE to ethene degradation resulted in higher *Dehalococcoides* abundance than without pretreatment. Subsequent field studies further supported this showing dechlorination of >95% TCE and VC. This strategy may be a viable option for the BIP as well, although studies to determine how well fermentative bacteria can survive in highly CHC-impacted sites would be required and may still necessitate the prior reduction in total CHC as discussed above. Alternatively, determining the receptiveness of biostimulated microcosms in this study to subsequent bioaugmentation may result in CHC degradation. The adjustment of pH and/or amendment of EVO both showed shifts in microbial abundances, largely to SRB, which may indicate the groundwater is more amenable to OHRB growth and bioremediation.

## 5 Conclusion

This study examined the ability of the physiochemical parameters and indigenous microbial community of a CHC impacted site to serve as predictive markers for natural attenuation or enhanced bioremediation. All but one site selected for this study failed to demonstrate degradation of any CHC by natural attenuation, while nine of the 21 sites showed some level of bioremediation following enhanced bioremediation. The main factors inhibiting bioremediation were complex and high CHC load. Elevated levels of chlorinated methanes proved the most inhibitory. Secondly, elevated levels of sulfate were associated with a lack of CHC degradation, likely driven by the co-enrichment of SRB which may outcompete OHRB for electron donors under high sulfate conditions. Finally, multiple sites were able to demonstrate CHC degradation despite the low quantity of indigenous microbial populations suggesting the sites were unfavorable to bioremediation—suggesting native microbial population is not indicative of whether a site can respond to enhanced bioremediation strategies.

## Data Availability

The datasets presented in this study can be found in online repositories. The names of the repository/repositories and accession number(s) can be found in the article/Supplementary material.
