# Supplementary material for: Bioremediation of complex chlorinated-solvent impacted groundwater
Source: Front Microbiol. 2026 Sep 17;17:1845514. doi: 10.3389/fmicb.2026.1845514 (PMC13627306; doi:10.3389/fmicb.2026.1845514)
Supplement: Supplementary file 1 [file Supplementary_file_1.DOCX]

**Bioremediation of complex chlorinated solvent impacted groundwater**

***Supplementary Figures S1-S8***

**Evan Landers^1^, Matt Lee^1^, Ranjith Rajendran^1^, Emma Harding^1^, Katherine Picott^2^, Mitra Parsa^1^, Michael Manefield^1*^**

^1^Department of Civil and Environmental Engineering, University of New South Wales, Sydney, New South Wales, Australia

^2^Chemical Engineering and Applied Chemistry, University of Toronto, Toronto, Ontario, Canada

*** Correspondence:**
Michael Manefield
Manefield@UNSW.edu.au

**Figure S1 – Bioaugmentation enrichment cultures**. Bacterial community compositions of bioaugmentation cultures used in this study A) DHB culture used for TCM respiration to DCM. B) DSB culture used for 1,2-DCA respiration to ethene. Analysis based on community composition at time of inoculation of bioaugmentation microcosms.

**Figure S2 – Impact of chlorinated solvent concentration on bacterial abundance.** Increasing concentrations of chlorinated solvents is associated with reduced bacterial abundance. Degree of impact on bacterial abundance is solvent specific. Blue regions highlight sub-inhibitory chlorinated solvent concentrations (x-axis) as determined by 16S rRNA gene abundance below 10^3^ copies/mL (y-axis).

**Figure S3 – Microbial activity markers.** Gene copy number of sulfate reductase (*dsrA*) as measure of sulphate reduction capacity by SRB and methyl-coenzyme M reductase (*mcrA*) as measure of methanogenesis capacity by methanogenic archaea determined by qPCR.

**Figure S4 – Respiration of 1,2-DCA in Unamended P1-14.** Culture P1-14 showed complete respiration of 1,2-DCA after 60 days. Data represents average of triplicate replicates for bioticcultures, abiotic represents a single culture. Error bars indicate standard deviation.

**Figure S5 – pH adjusted microcosms.** Time course changes in average 1,2-DCA and TCM concentrations of pH-adjusted microcosms (blue line) relative to abiotic controls (red dashed line). Blue shading indicates the active mean with the standard deviation. Significance as determined by adjusted p-value is indicated as follows: [.] p<0.1; [*] p<0.05; [**] p<0.01, [***] p<0.001. Points where solvent concentration in the experimental replicates exceeded abiotic control were not considered significant degradation.

**Figure S6 – EVO amended microcosms.** Time course changes in average 1,2-DCA and TCM concentrations of pH-adjusted and EVO amended microcosms (blue line) relative to abiotic controls (red dashed line). Blue shading indicates the active mean with the standard deviation. Significance as determined by adjusted p-value is indicated as follows: [.] p<0.1; [*] p<0.05; [**] p<0.01, [***] p<0.001. Points where solvent concentration in the experimental replicates exceeded abiotic control were not considered significant degradation.

**Figure S7 – Bioaugmented microcosms.** Time course changes in average 1,2-DCA and TCM concentrations of pH-adjusted, EVO amended, and bioaugmented (either DSB or DHB) microcosms (blue line) relative to abiotic controls (red dashed line). Blue shading indicates the active mean with the standard deviation. Significance as determined by adjusted p-value is indicated as follows: [.] p<0.1; [*] p<0.05; [**] p<0.01, [***] p<0.001. Points where solvent concentration in the experimental replicates exceeded abiotic control were not considered significant degradation.

**(Previous page) Figure S8 Replicates with substantial CHC degradation.** Replicates from microcosms which did not show significant differences based on the average concentration, but which showed >50% degradation of 1,2-DCA and/or TCM in one or more replicate. Timepoints at which these replicates passed this 50% threshold are demarcated with vertical bars and labelled with the replicate(s) code. **(A)** pH-Adjusted **(B)** EVO amended **(C)** Bioaugmented.
